# Retrograde access of the left atrium for pulmonary vein isolation using magnetic navigation after closure of an atrial septum defect

**DOI:** 10.1007/s12471-015-0701-x

**Published:** 2015-05-28

**Authors:** L. De Roeck, L. Riahi, S. Wijchers, D. Stockman, Y. De Greef, B. Schwagten

**Affiliations:** 1 University of Antwerp, Campus Drie Eiken, Universiteitsplein 1, 2610 Antwerp (Wilrijk), Belgium; 2 ZNA Middelheim Hospital, Lindendreef 1, 2020 Antwerp, Belgium

**Keywords:** Ablation, Magnetic navigation, Arrhythmia, Electrophysiology

## Abstract

Transseptal puncture is the most commonly used technique to perform electrophysiological procedures in the left atrium. This case report describes a pulmonary vein isolation in a patient with a paroxysmal atrial fibrillation, complicated by the presence of an oversized Amplatzer device (AGA Medical Corp., Golden Valley, MN). A retrograde approach using the magnetic navigation system (Niobe, Stereotaxis Inc., St Louis, USA) was performed, and showed to provide a feasible, safe and successful alternative for catheter ablation of cardiac arrhythmias in patients in whom the classic transseptal approach is impossible.

## Introduction

The unique capabilities of the magnetic navigation system (MNS) offer the possibility to perform ablations that would not be possible manually. We report the case of a retrograde transaortic approach to perform a pulmonary vein isolation (PVI).

**Fig. 1:**
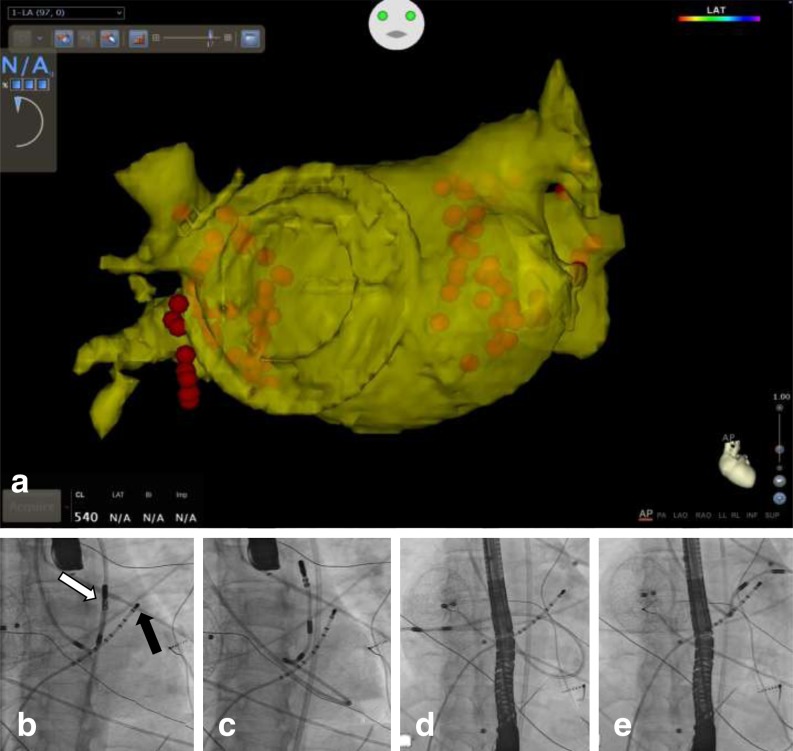
Retrograde pulmonary vein isolation. **a** Postprocedural CARTO electro-anatomic-CT merge map of the left atrium. The round deformation at the septum is the Amplatzer device and the *red dots* mark the ablation points. **b**–**e** *White arrow* indicates the ablation catheter, *black arrow* indicates the coronary sinus catheter. **b** Ablation catheter is positioned above the aortic valve. **c** Ablation catheter crosses the aortic valve retrogradely, passes through the left ventricle, crosses the mitral valve retrogradely and is positioned with its tip on the roof of the left atrium. **d** Ablation catheter is advanced into the right inferior pulmonary vein. **e** Ablation catheter is advanced into the left inferior pulmonary vein. In our centre, a transoesophageal echocardiogram probe is used during PVI procedures to enable monitoring for cardiac tamponade

 A 41-year-old female patient with highly symptomatic atrial fibrillation (AF) was referred to our department for PVI. Since she had undergone closure of an atrial septal defect with an oversized Amplatzer device (AGA Medical Corp., Golden Valley, MN) 4 years earlier (Figure 1), a classic transseptal puncture was considered to be a risky procedure and a retrograde approach using the MNS (Niobe, Stereotaxis Inc., St Louis, USA) was preferred.

## Methods

A preoperative computed tomography (CT) scan showed a common ostium for the right and left pulmonary veins (PVs). One venous access was achieved in the right femoral vein and a diagnostic decapolar catheter (Bard Electrophysiology, Lowell, MA, USA) was introduced through the femoral vein and positioned in the coronary sinus. The arterial access was gained through the right femoral artery using a 9F sheath (St. Jude Medical Inc., St. Paul, MN). A magnetically enabled, steerable tip ablation catheter (Navistar RMT Thermocool, Biosense Webster, Diamond Bar, CA, USA) was introduced through the femoral artery and was connected to the QuickCas for remote-controlled magnetic navigation. This catheter reached the left atrium via a retrograde route, passing through both the aortic and mitral valve (Figure 1). An electroanatomic map of the left atrium was made using the CARTO system (Biosense Webster, Diamond Bar, CA, USA). This map was merged with the CT scan. Using a sequential point-by-point antrum encirclement of the common ostia, the PVs were isolated. Bidirectional conduction block was proven on the basis of the Stereotaxis’ Bulls Eye technique. A radial collection of 16 points was taken within each PV by gradually changing the magnetic field. For each of these points, entry and exit block was shown: loss of PV potentials on the ablation catheter and failure to capture the LA by pacing (at 10 mA and 2 ms).

## Results

There were no complications during the procedure and the postablation recovery was unremarkable. The patient was discharged the next day, after transthoracic echocardiography, which excluded any valvular damage or pericardial effusion. No signs of arrhythmia recurrence was revealed after 1-year follow-up.

## Discussion

Transseptal puncture is the most commonly used technique to perform electrophysiological procedures in the left atrium. Retrograde approach to the left atrium using magnetic navigation and a remote-controlled catheter provides an interesting alternative for PVI, especially when a classic transseptal puncture cannot be performed [1]. The magnetic navigation system, with its soft catheter shaft, provides a stable catheter position and an extreme manoeuvrability even within the most complex anatomical conditions that cross the limits of conventional manually steered catheters [2, 3]. The use of a lasso catheter to check the PVs can be replaced by the Stereotaxis’ Bulls Eye technique.

### Funding

None

### Conflict of interests

None declared
